# Peptides/Proteins Encoded by Non-coding RNA: A Novel Resource Bank for Drug Targets and Biomarkers

**DOI:** 10.3389/fphar.2018.01295

**Published:** 2018-11-13

**Authors:** Song Zhu, Jizhong Wang, Yutian He, Nan Meng, Guang-Rong Yan

**Affiliations:** ^1^Biomedicine Research Center, The Third Affiliated Hospital of Guangzhou Medical University, Guangzhou, China; ^2^Key Laboratory of Protein Modification and Degradation, Guangzhou Medical University, Guangzhou, China

**Keywords:** long non-coding RNA (lncRNA), circular RNA (circRNA), primary miRNA (pri-miRNA), small ORF, small peptide, drug target, biomarker

## Abstract

Non-coding RNAs (ncRNAs) are defined as RNA molecules that do not encode proteins, but recent evidence has proven that peptides/proteins encoded by ncRNAs do indeed exist and usually contain less than 100 amino acids. These peptides/proteins play an important role in regulating tumor energy metabolism, epithelial to mesenchymal transition of cancer cells, the stability of the c-Myc oncoprotein, and the ubiquitination and degradation of proliferating cell nuclear antigen (PCNA). These peptides/proteins represent promising drug targets for fighting against tumor growth or biomarkers for predicting the prognosis of cancer patients. In this review, we summarize the characteristics of peptides/proteins that have recently been identified as putative ncRNA translation products and their outlook for small molecule peptide drugs, drug targets, and biomarkers.

## Introduction

Non-coding RNAs (ncRNAs) comprise a class of RNA nucleic acid molecules that are transcribed from DNA but do not encode proteins (Guttman et al., 2013). However, ncRNAs are involved in many diseases (Cho, 2011; Esteller, 2011; Hu et al., 2015; Zhou et al., 2016; Nan et al., 2017; Luo et al., 2018) and utilize a variety of mechanisms to participate in gene regulation, including DNA methylation, chromatin modifications, transcriptional regulation, mRNA splicing, translation control, RNA stability, and so on (Muro et al., 2011; Pelechano and Steinmetz, 2013; Xing et al., 2014; Huang and Shan, 2015).

**FIGURE 1:**
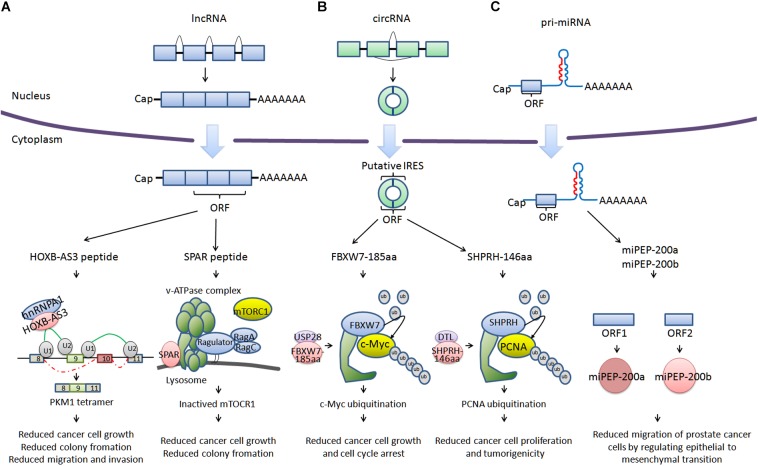
Cancer-related peptides/proteins encoded by ncRNAs. **(A)** Cancer-related peptides encoded by lncRNA. HOXB-AS3 is encoded by the lncRNA *HOXB-AS3*. HOXB-AS3 regulates tumor energy metabolism by antagonizing hnRNP A1, mediating PKM splicing. SPAR is encoded by *LINC00961.* SPAR interacts with the lysosomal v-ATPase to negatively regulate mTORC1 activation. **(B)** Cancer-related proteins encoded by circRNA. FBXW7-185aa is encoded by circ-*FBXW7*. FBXW7-185aa regulates the stability of the c-Myc oncoprotein. SHPRH-146aa is encoded by circ-*SHPRH*. SHPRH-146aa mediates the ubiquitination and degradation of PCNA. **(C)** Cancer-related proteins encoded by pri-miRNA. miPEP-200a and miPEP-200b are encoded by pri-miRNA (miR-200a and miR-200b). miPEP-200a and miPEP-200b affect the epithelial to mesenchymal transition of prostate cancer cells.

For a long time, there has been widespread recognition that ncRNA are unable to encode proteins (Guttman et al., 2013). However, with the development of deep ribosome profiling sequencing (Ribo-Seq) technology, mass spectrometry and algorithms including fragment length organization similarity score (FLOSS; Ingolia et al., 2014), ORF-RATER (Fields et al., 2015), and Ribo taper (Calviello et al., 2016), a subset of ncRNA have been identified to be able to encode peptides (<100 amino acids) or proteins, such as HOXB-AS3 (Huang et al., 2017), SPAR (Matsumoto et al., 2017), FBXW7-185aa (Yang et al., 2018), SHPRH-146aa (Zhang et al., 2018), miPEP-200a, and miPEP-200b (Fang et al., 2017; Figure 1). HOXB-AS3 and SPAR are encoded by long non-coding RNA (lncRNA). HOXB-AS3 plays an important role in regulating tumor energy metabolism, and SPAR interacts with the lysosomal v-ATPase to negatively regulate mTORC1 activation. FBXW7-185aa and SHPRH-146aa are encoded by circular RNA (circRNA), regulating the stability of the c-Myc oncoprotein and mediating ubiquitination and degradation of proliferating cell nuclear antigen (PCNA). miPEP-200a and miPEP-200b are encoded by primary miRNA (pri-miRNA). miPEP-200a and miPEP-200b affect epithelial to mesenchymal transition of cancer cells. We describe a bank of biologically functional peptides/proteins encoded by lncRNA, circRNA, and pri-miRNA. The biological activity of ncRNA encoded peptides/proteins may provide possibilities for developing new therapies for current refractory and other diseases.

Peptides/proteins encoded by ncRNAs represent promising drug targets for fighting against tumor growth or biomarkers for predicting the prognosis of cancer patients. In this review, we summarize the characteristics of peptides/proteins encoded by ncRNA and their outlook for small molecule peptide drugs, drug targets, and biomarkers.

## Peptides/Small Proteins Encoded by NCRNA as Promising Candidates in Antitumor Drugs

### Advantages and Development Status of Anti-tumor Peptide/Small Protein Drugs

It is generally known that some peptide/protein drugs (such as antibacterial peptides) have been widely used in the field of animal medicine and have achieved good results (Brogden et al., 2003). In addition, the use of peptides/small protein drugs has a long successful history in the treatment of human disease, for instance, insulin for the treatment of diabetes (Kaur et al., 2018). Some anti-tumor peptides/small proteins such as mifamurtide (Brosa et al., 2014), interferon-γ (INF-γ; Berek, 2000), and interleukin-2 (IL-2; Liang et al., 2012), have been used in clinics and achieved a certain curative efficacy. Although chemotherapy is the major treatment method for cancer, it has fierce side effects, including the insufficient uptake of drugs in tumor cells, non-specific cytotoxicity of chemotherapeutic drugs and the rapid emergence of drug resistance, which largely limit clinical application (Gandhi et al., 2014; He et al., 2016). Compared with traditional chemotherapy agents, peptide and protein drugs possess unique advantages, including high specificity and activity, low immunogenicity and less cytotoxicity to normal tissues (Leader et al., 2008). Therefore, peptide and protein therapeutics have emerged as crucial approaches in recent clinical cancer treatment. The anti-cancer mechanisms of peptides and proteins are mainly divided into two types: one is usually related to direct induction of apoptosis in tumor cells via specific pathways, and the other is related to direct inhibition of tumor cell growth through targeting tumor angiogenesis or stimulating an immune response (Vaishya et al., 2015; Redington et al., 2017). Some protein drugs, such as tumor necrosis factor alpha (TNF-α), INF-γ, and IL-2, have been widely used in clinical cancer treatment (Berek, 2000; Calzascia et al., 2007; Liang et al., 2012). Nevertheless, despite TNF-α, INF-γ, and IL-2 possessing anti-tumor potency, the systemic clinical applications of high doses of these drugs are severely limited due to their systemic toxicities, for instance, inflammatory response, and underlying cardiotoxicity (Ashkenazi et al., 1999; Berek, 2000; Liang et al., 2012). Therefore, it is necessary and urgent to continue looking for higher efficiency and lower toxicity anti-tumor drugs.

### Application Prospect of Peptides/Small Proteins Encoded by lncRNAs in the Treatment of Tumors

Only 2% of the human genome is able to code for proteins or peptides, and the rest is actively transcribed into ncRNAs (Fang et al., 2017). Surprisingly, a limited number of “ncRNAs” have recently shown the ability to encode proteins or peptides. *HOXB-AS3* was annotated as lncRNA in the NCBI and lncRNA databases. Our research team has recently discovered that the lncRNA *HOXB-AS3* could actually encode a conserved 53-aa peptide, called HOXB-AS3 peptide (Huang et al., 2017). *In vitro* studies showed that HOXB-AS3, rather than its lncRNA, inhibited proliferation, migration, invasion, and colony formation of colon cancer cells (CRC) via antagonizing hnRNP A1 protein (Huang et al., 2017). More importantly, the HOXB-AS3 peptide clearly impaired the *in vivo* growth of CRC xenografts and decreased the number and size of lung metastatic nodules (Huang et al., 2017). Therefore, HOXB-AS3 has great potential for the treatment of colon cancer via antagonizing hnRNP A1. Moreover, a conserved lncRNA *LINC00961*, encodes a novel peptide, called “small regulatory peptide of amino acid response” (SPAR). SPAR locates at the late endosome/lysosome and interacts with the lysosomal v-ATPase to negatively regulate mTORC1 activation (Matsumoto et al., 2017).

### Application Prospect of Peptides/Small Proteins Encoded by circRNAs in the Treatment of Tumors

Very few circRNAs have been shown to encode small proteins with antitumor activity based on small-ORFs. Zhang et al. (2018) reported that a novel antitumor protein, SHPRH-146aa, was encoded by the circular RNA *SHPRH* and was driven by internal ribosome entry site (IRES) elements. SHPRH-146aa was able to protect full-length SHPRH from degradation by the ubiquitin-proteasome. Stabilized SHPRH inhibited glioblastoma cell proliferation and tumorigenicity via sequentially ubiquitinating PCNA as an E3 ligase (Zhang et al., 2018). In addition, Yang et al. (2018) also found that a novel 21-kDa protein, named FBXW7-185aa, was encoded by the circular RNA *FBXW7*. The FBXW7-185aa protein can reduce the half-life of the c-Myc oncoprotein, which is known to regulate the transcription of numerous genes and pathways, via antagonizing ubiquitin carboxyl-terminal hydrolase 28 (USP28) induced c-Myc stabilization in glioma cells (Yang et al., 2018). Therefore, the FBXW7-185aa protein can inhibit the proliferation and cell cycle acceleration of malignant glioma cells by downregulating the protein expression of c-Myc (Yang et al., 2018).

### Application Prospect of Peptides/Small Proteins Encoded by pri-miRNAs in the Treatment of Tumors

Impressively, Fang et al. (2017) found that two proteins (miPEP-200a and miPEP-200b) were encoded by miR-200a and miR-200b, respectively. The experiment results indicated that miPEP-200a and miPEP-200b suppressed the migration of prostate cancer cells through regulating the epithelial to mesenchymal transition of tumor cells.

### Application of Peptide/Small Protein Drugs Encoded by ncRNA

The ideal drug not only has specificity and pharmacological activity, but can also reach the target site to play a role. Whether these cancer-suppressive peptides/small proteins (SPAR, HOXB-AS3, FBXW7-185aa, SHPRH-146aa, miPEP-200a, and miPEP-200b) encoded by ncRNAs, are secreted into human serum remains unknown. Whether they can enter tumor cells through transporters on the cell membrane and play an inhibitory role remains unknown. Due to the potential developmental value and clinical applications, these problems will become a hot topic of research. In the past few decades, nanoscale carriers have become one of the key strategies for enhancing cancer treatment, which is attributed to the markedly enhanced tumor accumulation of drugs, prolonged blood circulation of the drug-loaded nanoparticles, and improved intracellular delivery efficiency (Elsabahy and Wooley, 2012; He et al., 2016). Therefore, if these peptides/small proteins have a very short half-life *in vivo* and are difficult to pass the biofilm barrier, they could be wrapped in nanomaterials to avoid being quickly metabolized and be delivered to tumor cells through nanoparticles to play an anti-cancer role. Moreover, these peptides/small proteins could interact with chemical drugs to treat tumors through nanoscale codelivery systems. Recombinant human adenovirus-p53 injection has become a new method for the clinical treatment of cancer. After the coding sequence of these peptides/small proteins is recombined with adenovirus, it could be injected into patients to treat tumors.

## Peptides/Small Proteins Encoded by NCRNA as Promising Cancer Drug Targets

### Targeted Therapy

Targeted therapy refers to a type of treatment that uses drugs or other substances to attack specific targeted molecules (e.g., certain enzymes, proteins, DNA, RNA or other molecules), thereby maximizing efficacy and minimizing toxicity (Sawyers, 2008). This is also called “molecularly targeted therapy” and “precision medicine.” The purpose of targeted therapy in cancer is to restrict the growth and survival of cancer cells without injury to normal cells. In view of tumor heterogeneity, several types of targeted therapies, including monoclonal antibodies (e.g., Rituximab and Infliximab) (Maifrede et al., 2018), angiogenesis inhibitors (e.g., Pemetrexed and FGFR inhibitors) (Rojas et al., 2016), hormone therapies (e.g., Palbociclib) (Lynce et al., 2018), immune therapies (e.g., Durvalumab) (Raja et al., 2018), and rapamycin target inhibitor (e.g., Everolimus) (Kornblum et al., 2018), have been approved for cancer treatment. Additionally, tumor suppressor have also been studied as targets for targeted therapies in cancer. An example is the most important tumor suppressor protein, p53, which is associated with carcinogenesis by missense mutation (Brosh and Rotter, 2009). It was reported that 96.7% of high-grade serous ovarian carcinoma (HGSOC) cases contain pathogenic TP53 mutations (Ahmed et al., 2010). More recently, Soragni et al. (2016) designed a peptide inhibitor of p53 amyloid aggregation, ReACp53, which penetrated cells and restored the p53 function of tumor suppression in HGSOCs.

### Application of ncRNA Encoded Peptides/Proteins as Promising Therapy Targets

ncRNA encoded peptides/proteins (HOXB-AS3, FBXW7-185aa, SHPRH-146aa, miPEP-200a, and miPEP-200b) have been proved to suppress tumorigenesis, which has enriched the research of ncRNAs in cancer development. Whether or not these tumor suppressor ncRNAs present mutations, like TP53 does, in tumors is still unknown. Strategies for rescuing or strengthening the function of tumor suppressor peptides/proteins, including vaccination with synthesized peptides or viral vector vaccines that encode relevant peptides sequences for cancer therapies, are now in development (Efremova et al., 2017; Radvanyi, 2018). Meanwhile, in the human genome, increasing evidence suggests that numerous ncRNAs are functional and play pivotal roles in many aspects of biology (Esteller, 2011). Maybe there are some hidden oncopeptides/oncoproteins encoded by ncRNAs that need to be identified; these hidden oncopeptides/oncoproteins may be exploited as novel targets for targeted therapies in cancer.

## Peptides/Proteins Encoded by NCRNA as Promising Cancer Biomarkers

### Biomarkers in Clinical Detection

With the advancement of treatment modalities, the survival rate and quality of life for early-stage cancer patients have been improved. However, due to the lack of specific symptoms and signs of early-stage cancer, and limited by cancer heterogeneity, only a portion of cancer patients are diagnosed early. Most cancer patients experience tumor recurrence and metastasis at regional or distant sites, which seriously shortens their survival time and greatly diminished quality of life. It is obvious that cancer bring devastating effects on patients and their families, which is a tremendous burden on society. Bray estimated there would be 22.2 million new cancer cases in 184 countries by 2030 (Bray et al., 2012). Cancer has been a severe challenge to society, and the early detection of cancer is highly important for clinical work.

It has been widely recognized that cancer is a very heterogeneous disease, and implementing precision medicine for patients is widely accepted. The molecular subtype of cancer provides us a new perspective: for different molecular subtypes, we can develop different, appropriate therapies and monitor the disease progression in the most suitable way. For instance, patients with triple-negative breast cancer (TNBC) have an increased risk of death compared with women with other types of breast cancer (Mayer et al., 2014). Gene expression analyses recently showed that TNBC has six distinct subtypes, each displaying a unique biology and showing distinct responses to standard treatment (Lehmann et al., 2011; Masuda et al., 2013). It is urgent to develop more biology-specific cancer biomarkers so that patients can receive the most appropriate therapy. Developing more suitable biomarkers for different molecular typing of tumors will be an inevitable biological challenge for cancer treatment. Therefore, biology-specific cancer biomarkers urgently need to be discovered and developed.

### Peptides/Proteins Encoded by ncRNA as Promising Cancer Biomarkers

#### HOXB-AS3 Encoded by lncRNA Is a Potential Prognostic Biomarker

Our research team reported that the HOXB-AS3, encoded by the lncRNA *HOXB-AS3*, has been proven to play an important role in the development of cancer metabolism reprogramming. In mice, the *in vivo* growth of CRC xenografts with *HOXB-AS3* ORF and 5′ UTR-ORF stably transfected cells was clearly impaired, including inhibition of tumor growth and lung metastasis. The HOXB-AS3 levels were decreased in primary CRC tissues compared with their paired non-tumoral tissues. Comparing the correlations between the level of HOXB-AS3 and clinic pathological features in 90 CRC cases, we found that patients with the clinical stage between IIB to IV have significantly lower levels of HOXB-AS3. The mean overall survival time for CRC patients with high levels of HOXB-AS3 was 1.6 times that of CRC patients with low levels of HOXB-AS3. Kaplan-Meier survival analyses revealed that patients with lower HOXB-AS3 levels were at increased risk of CRC-related death. Therefore, the low level of the HOXB-AS3 was correlated with a poor prognosis in CRC patients (Huang et al., 2017).

#### The Potential of Peptides/Proteins Encoded by circRNAs as Cancer Biomarkers

Nu Zhang’s research team reported that circ-*FBXW7* can encode a 21-kDa protein, called FBXW7-185aa, which is assumed as a prognostic marker for glioblastoma. The mice injected with glioblastoma multiforme cells that stably overexpressed circ-FBXW7 had longer survival times and exhibited a much lower tumorigenicity than mice injected with the corresponding control cells. In 38 pairs of glioblastoma samples compared with their paired tumor-adjacent tissues, the expression of FBXW7-185aa was obviously decreased. The protein expression was investigated to demonstrate that glioblastoma patients with higher FBXW7-185aa expression had an increased total survival time (Yang et al., 2018).

Another study focused on SHPRH-146aa, which is encoded by circ-*SHPRH*, and showed that the SHPRH-146aa expression was significantly downregulated in 60 glioblastoma samples compared with their adjacent normal tissues. The subcutaneous injection with SHPRH-146aa stably overexpressing cells resulted in substantially lower tumorigenicity compared with the injection of the corresponding control cells in mice. Cancer patients with higher SHPRH-146aa expression had a longer survival time than those with lower SHPRH-146aa expression in glioblastoma patient samples. The result suggested SHPRH-146aa is a prognostic marker for glioblastoma in clinics (Zhang et al., 2018).

#### The Potential of Peptides/Proteins Encoded by pri-miRNAs as Cancer Biomarkers

The primary microRNA, miR-200a and miR-200b, that encode proteins (miPEP-200a and miPEP-200b) can inhibit the migration of prostate cancer cells, and it was observed that low-level expression of miPEP-200a and miPEP-200b were associated with poor survival outcome in cancer patients. This evidence indicated that miPEP-200a and miPEP-200b have the potential to be used as diagnostic and prognostic markers (Fang et al., 2017).

### The Expectation of Peptides/Proteins Encoded by ncRNA Used as the Cancer Biomarkers

Accumulating evidence has shown that peptides/proteins encoded by ncRNA represent a promising source for prognostic biomarkers. Although the differential expression and prognostic correlation of the five peptides/proteins HOXB-AS3, SHPRH-146aa, FBXW7-185aa, miPEP-200a and miPEP-200b in cancer have been confirmed by the IHC analysis of paraffin sections of tumor tissues and western blot analysis of protein samples, we do not see a clear report that the peptides/proteins encoded by ncRNA are detected in body fluid (including blood, serum, urine, chest fluid, etc.). If these biology-specific peptides/proteins can be found in body fluid, the widespread progress of using them as cancer biomarkers in clinical applications will be greatly promoted. The tremendous research space of peptides/proteins encoded by ncRNAs used as cancer biomarkers will encourage researchers to continuously devote great effort in this field so that they can meet the requirements of being reliable, cost-effective, and capable of precise detection and monitoring.

## Conclusion

ncRNA-encoded peptides/proteins open up a new field. The peptides/proteins mentioned in this review affect multiple steps in the development of tumorigenesis. These peptides/proteins regulate tumor energy metabolism, the stability of oncoproteins and the epithelial to mesenchymal transition of cancer cells. They are the new stars of the future drug targets for fighting against tumor growth or may be used as biomarkers for predicting the prognosis of cancer patients.

However, the ncRNA-encoded peptides/proteins that have been discovered so far are only the beginning, and those that are undiscovered will provide a wealth of opportunities for small molecule peptide drugs, drug targets, and biomarkers. Currently, whether these ncRNA-encoded peptides/proteins can be used in clinical practice requires a large sample of clinical studies. Follow-up treatment effects of ncRNA-encoded peptides/proteins and detecting these as biomarkers in more samples will be needed.

## Author Contributions

G-RY conceived the general idea. SZ, JW, YH, and NM wrote the first draft. G-RY revised the manuscript. All authors read and approved the final manuscript.

## Conflict of Interest Statement

The authors declare that the research was conducted in the absence of any commercial or financial relationships that could be construed as a potential conflict of interest.
